# Case Report: Successful endovascular treatment of acute type A aortic dissection

**DOI:** 10.3389/fcvm.2023.1299192

**Published:** 2023-11-16

**Authors:** Leonard Pitts, Roland Heck, Matteo Montagner, Adam Penkalla, Markus Kofler, Volkmar Falk, Jörg Kempfert, Semih Buz

**Affiliations:** ^1^ Department of Cardiothoracic and Vascular Surgery, Deutsches Herzzentrum der Charité (DHZC), Berlin, Germany; ^2^ Charité—Universitätsmedizin Berlin, Corporate Member of Freie Universität Berlin and Humboldt-Universität zu Berlin, Berlin, Germany; ^3^ DZHK (German Centre for Cardiovascular Research), Partner Site, Berlin, Germany; ^4^ Department of Health Sciences and Technology, Translational Cardiovascular Technologies, Institute of Translational Medicine, Swiss Federal Institute of Technology (ETH), Zurich, Switzerland

**Keywords:** aortic dissection, type A, TEVAR, endovascular, ascending aorta

## Abstract

**Introduction:**

Open surgical repair remains the current gold standard for the treatment of acute type A aortic dissection. However, especially elderly patients with relevant comorbidities who are deemed unfit for open surgery may benefit from a minimally invasive endovascular approach.

**Methods:**

We report a case of an 80-year-old male with retrograde acute type A aortic dissection and peripheral malperfusion after receiving thoracic endovascular aortic repair due to thoracic aortic aneurysm. Our individualized endovascular approach consisted of left carotid-subclavian bypass, proximal extension of thoracic endovascular aortic repair using a covered stent graft and a single covered stent graft for the ascending aorta in combination with an uncovered stent for the aortic arch.

**Results:**

Postoperative computed tomographic angiography demonstrated excellent outcome with no signs of endoleak or patent false lumen. Follow-up after 3.5 years showed a stable result with no signs of stent failure or dissection progress. No aortic re-interventions were needed in the further course.

**Discussion:**

An individualized endovascular approach may be justified for acute type A aortic dissection in elderly patients with high surgical risk if performed in specialized aortic centers. Additional short-length stent graft devices are needed to address the anatomical challenges of the ascending aorta. For enhanced remodeling of the dissected aorta, the use of an additional uncovered stent may be advisable.

## 1. Introduction

Acute type A aortic dissection (ATAAD) is a possibly lethal event that demands urgent surgical repair according to current guidelines (1, 2). Though surgical strategies have been developed continuously, mortality and morbidity are still high (3). Perioperative complications such as stroke are not uncommon and may influence the patients’ outcome significantly (4, 5). Especially in elderly patients, malperfusion and advanced age may lead to significant increase in perioperative risk, possibly even reconsidering the decision for standardized open repair (6). Therefore, offering the possibility of endovascular instead of open surgical treatment for these patients to reduce surgical trauma may be of great interest. Compared to standardized interventional treatment of acute type B aortic dissection by thoracic endovascular aortic repair (TEVAR), an interventional approach for ATAAD comes along with major challenges (2). Due to the possible involvement of the aortic root and aortic valve as well as the coronary arteries and branch vessels of the aortic arch, a stent-based “TEVAR-like” approach is accompanied by substantial technical difficulties and may even be harmful. Various prostheses are available on the market to perform standardized TEVAR of the descending thoracic aorta, e.g., GORE C-TAG® or TAG® (GORE®, Flagstaff, AZ, USA), ZENITH TX2 and Alpha (Cook®, Bloomington, IN, USA), Valiant Captiva (Medtronic®, Dublin, Ireland), or Relay® (Bolton, Barcelona, Spain; now Terumo®, Inchinnan, UK) to name the most popular ones. Thereby, in case of TEVAR for thoracic aortic aneurysm, oversizing of the stent graft is an important key point and should at least exceed the diameter of the landing zones by 10%–15% of the reference aortic diameter (7). In case of type B aortic dissection, oversizing should almost be avoided and may even lead to a higher risk for retrograde dissection if the oversizing rate is greater than 5% (8). Transferring these experiences for the treatment of the ascending aorta, especially in case of ATAAD, should be performed with caution. However, we believe that under certain circumstances, the evaluation of interventional treatment options for ATAAD may be justified to avoid open central repair in elderly patients with several comorbidities and consecutive high perioperative risk. Therefore, we report a case of an 80-year-old male presenting with retrograde ATAAD and consecutive successful endovascular treatment.

## 2. Case description

### 2.1. Patient information

**Figure 1:**
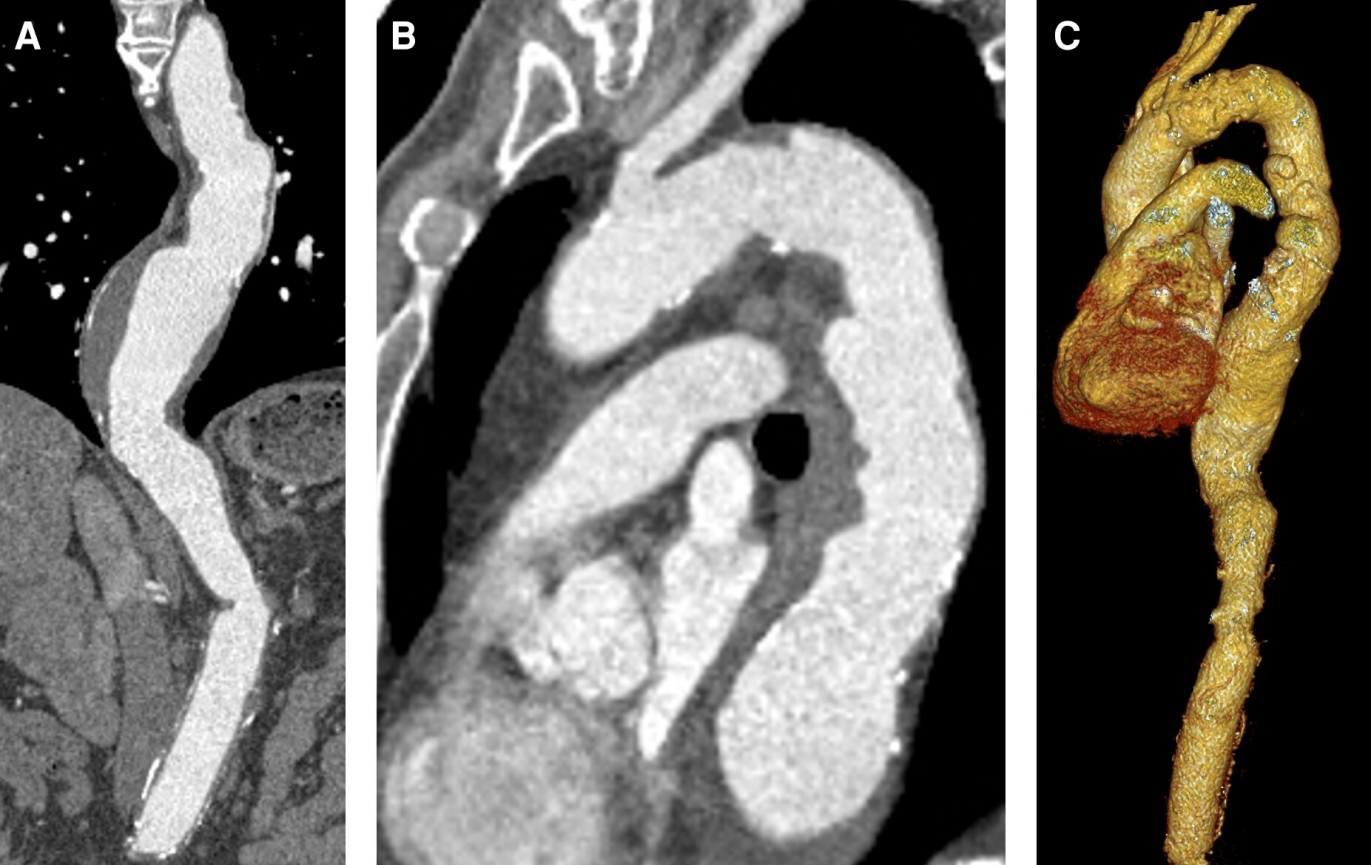
Preoperative CTA. (**A**) Descending aortic aneurysm with 63 mm diameter. (**B**) Proximal landing zone distal to the left subclavian artery (30 mm). (**C**) 3D reconstruction.

An 80-year-old male patient underwent elective computed tomographic angiography (CTA) scan for checkup of a known thoracoabdominal aneurysm. Nine years before, he received open surgical repair of juxtarenal abdominal aortic aneurysm with reimplantation of the left renal artery. Results of the recent CTA (SOMATOM Definition Flash, Siemens®, Erlangen, Germany) are depicted in Figure 1 and showed a thoracic aortic aneurysm with 63 mm of the descending aorta. According to the current guidelines, TEVAR was recommended to prevent aortic dissection or rupture (2). Therefore, he was referred to a local vascular surgery department for TEVAR. Proximal landing zone diameter distal from the left subclavian artery was estimated to be 30 mm. At this point of time, the patient was free of symptoms. Due to the involvement and reimplantation of the left renal artery, the left kidney was already atrophic for several years, resulting in chronic renal failure stage 3b with a glomerular filtration rate of 45 ml/min. In addition, he suffered from long-term arterial hypertension with fourfold antihypertensive medication, non-insulin-dependent type 2 diabetes and dyslipidemia, both treated with oral medication, as well as an adequate substituted hypothyroidism. There was no anamnestic evidence of genetic or hereditary aortic diseases as well as similar familiar cases.

The patient was prepared for TEVAR in a standard fashion via right femoral artery under general anesthesia. After releasing the thoracic stent graft (Valiant Navion™ 37/37/255 mm, Medtronic®, Dublin, Ireland), which included >20% proximal oversizing, perioperative angiography showed retrograde acute aortic dissection at the proximal edge of the TEVAR prosthesis involving the aortic arch and the ascending aorta. The patient was quickly referred to CTA, which confirmed the diagnosis of retrograde ATAAD. He was transferred stable and uneventfully to our clinic for further treatment.

### 2.2. Clinical findings

On admission, the patient was intubated, mechanically ventilated, and sedated. Pupils were small, equally round, and reactive to light. He was hemodynamically stable without any inotropic medication in normal frequent sinus rhythm; the lungs were ventilated and clear to auscultation bilaterally. The abdomen was soft and normoactive bowel sounds were present in all four quadrants. Diuresis was sufficient. The extremities were warm and peripheral pulses were palpable except for the right leg, which was cold and livid, indicating the presence of peripheral malperfusion. Hemoglobin was 11 g/dl and lactate 7 mg/dl; pulmonary function was not impaired. At this point of time, no clinical evidence of further systemic or localized malperfusion except for the right leg was present, resulting in class Penn Ab according to the current Penn classification (9).

### 2.3. Diagnostic assessment

**Figure 2:**
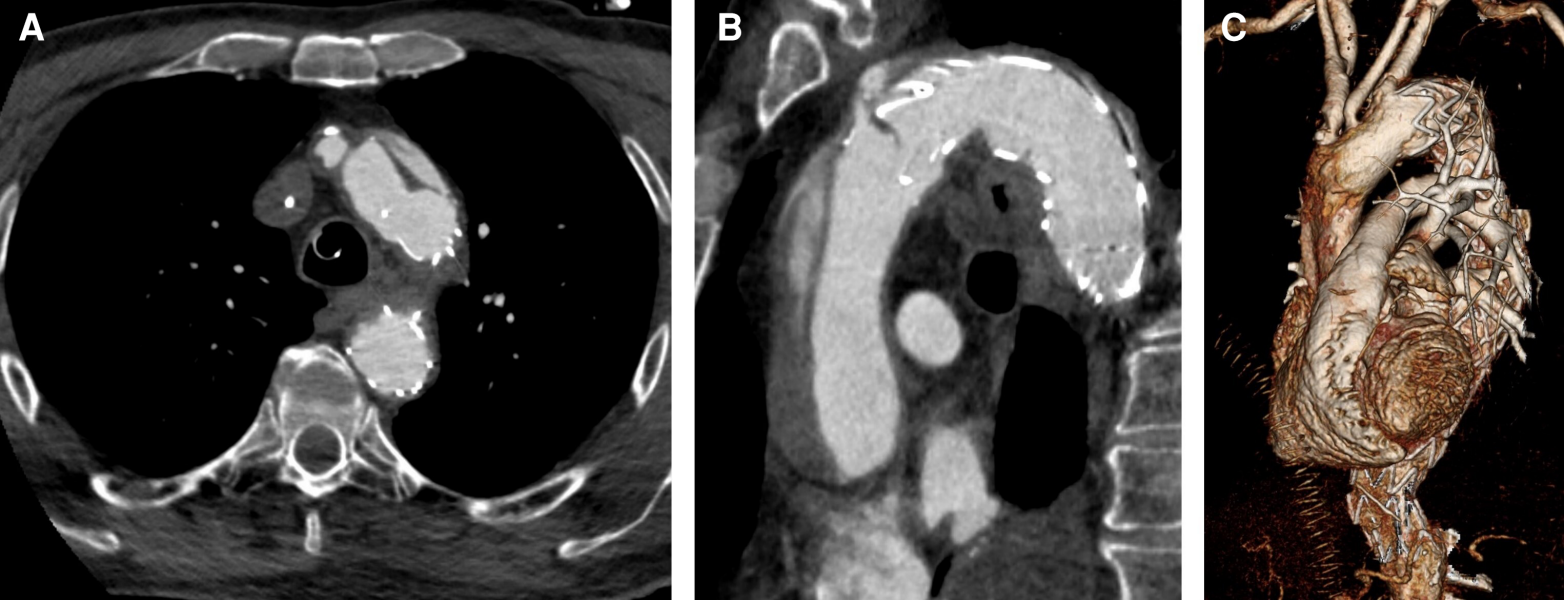
CTA after TEVAR and consecutive retrograde ATAAD. (**A**) Entry proximal of TEVAR stent graft. (**B**) Dissection involving the ascending aorta and the aortic arch. (**C**) 3D reconstruction.

After analyzing the CTA, iatrogenic ATAAD with entry location at the proximal edge of the TEVAR prosthesis in the descending aorta was confirmed, most likely caused by the tip of the stent. Entries in the aortic arch or the ascending aorta could not be identified. According to the new TEM classification by Sievers et al., this resulted in “A, E3, M3+” (A = type A dissection according to Stanford, E3 = entry in the descending aorta, M3+ = peripheral malperfusion with clinically relevant malperfusion) (10). Landing zone length of the ascending aorta was 72 mm, proximal landing zone diameter was 33 mm, and distal landing zone diameter was 28 mm. Preoperative CTA (SOMATOM Definition Flash, Siemens®, Erlangen, Germany) is shown in Figure 2. Imaging of the intracranial vessels was not available.

Considering the advanced age as well as extensive comorbidities, we decided to perform complete endovascular treatment in combination with surgical left carotid-subclavian bypass. Thereby, left carotid-subclavian bypass was performed to reduce the potential risk of spinal cord injury or cerebrovascular accident.

### 2.4. Therapeutic intervention

Prior to surgery, a 7 Fr sheath via left internal jugular vein was inserted in addition to the pre-existing 5 lumen central venous line and arterial access line in left radial artery. Near infrared spectroscopy (NIRS) electrodes were placed bilaterally on the forehead. General anesthesia was continued using propofol and sufentanil. The patient was placed in a supine position prior to standard sterile draping. After application of 2 g cefazoline and team timeout, a skin incision was made parallel to the left clavicle on the neck as access for carotid-subclavian bypass. After cutting the platysma, careful exposition of the jugular vein, the common carotid artery, and the vagus nerve was performed. Then, the left subclavian artery was exposed. After heparin administration, the vessel was partially clamped and incised longitudinally. A Dacron graft (FlowNit® Bioseal, 8 mm, Artivion®, Hechingen, Germany) was anastomosed end-to-side with 5–0 prolene. The same procedure was carried out for proximal anastomosis to the left common carotid artery after careful placement of a distal and proximal clamp. NIRS showed stable and equal values during the whole procedure. After successful de-airing, the circulation was released. For further angiography, a 5 Fr sheath was placed in the prosthesis after application of a purse-string suture followed by a diagnostic pigtail which was placed in the ascending aorta.

After successfully performing the carotid-subclavian bypass, a 2-cm skin incision was made in the right groin area and the right common femoral artery was punctured percutaneously. Two endovascular closure systems (ProGlide™, Abbott® Cardiovascular, Plymouth, MA, USA) were inserted and prepared for the end of the procedure. Then, a 16-Fr sheath was inserted followed by a flexible wire (Radifocus™, Terumo®, Inchinnan, UK) to pass the aortic valve for pigtail catheter-guided insertion of a pre-bent double curved wire (Safari™, Boston Scientific®, Marlborough, MA, USA), which was placed in the left ventricle. In addition, a temporary pacing electrode was placed in the right ventricle through the right femoral vein. After angiographic control of catheter position and visualization of true and false lumen, the 16-Fr sheath was removed and the stent graft delivery system (Relay® Plus NBS 34/34/109 mm, Terumo®, Inchinnan, UK) was inserted until reaching the aortic arch. After carefully placing the edge of the stent graft delivery system close to the branch of the left common carotid artery (Zone 2), rapid pacing was performed (180/min) and the stent was released. Angiographic control showed adequate expansion and stent position as well as sufficient perfusion of the left common carotid artery. As next step, another thoracic stent graft delivery system (Valiant Navion™ 37/37/55 mm, Medtronic®, Dublin, Ireland) was prepared and inserted until reaching the ascending aorta. Angiographic control was performed precisely to visualize both coronary ostia and the brachiocephalic trunk. Thereby, a 6-Fr pigtail (Boston Scientific®, Marlborough, MA, USA) was parked at the non-coronary cusp considering the radiographic angle to support the identification of the landing zone and avoid protrusion of the stent graft into the coronary arteries. After exact positioning between the coronary ostia and the brachiocephalic trunk, the stent graft was released during rapid pacing (180/min). For further stabilization and aortic remodeling, analogous to the PETTICOAT technique (provisional extension to induce complete attachment), an additional uncovered nitinol stent (E-XL™ 36/07 mm, formerly JOTEC® GmbH, now Artivion®, Hechingen, Germany) was inserted into the aortic arch to connect both stent grafts (11). Angiographic and perioperative transesophageal echocardiography showed satisfactory results with adequate positioning and successful elimination of false lumen flow. The stent graft delivery system including the sheath was removed, and endovascular vessel closure was performed by using the sutures prepared by ProGlide™. Then, the pigtail catheter and sheath were removed from the subclavian-carotid bypass graft. Finally, a redon drainage was placed and the wound was closed in layers finishing with intracutaneous skin suture.

**Figure 3:**
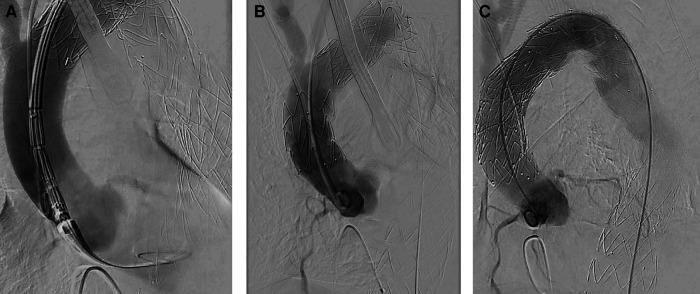
Intraoperative angiography. (**A**) Aortic dissection after releasing TEVAR stent for proximal expansion. (**B**) Aortic dissection after stenting of the ascending aorta and parking of a pigtail catheter in the non-coronary cusp. (**C**) Postoperative result after placing an uncovered stent in the aortic arch (“Reverse PETTICOAT”).

Perioperative transesophageal echocardiography revealed preserved biventricular function, no relevant aortic insufficiency, or any wall motion abnormalities. No persistent flow could be detected in the false lumen. Intraoperative angiographic steps are shown in Figure 3.

### 2.5. Follow-up and outcomes

**Figure 4:**
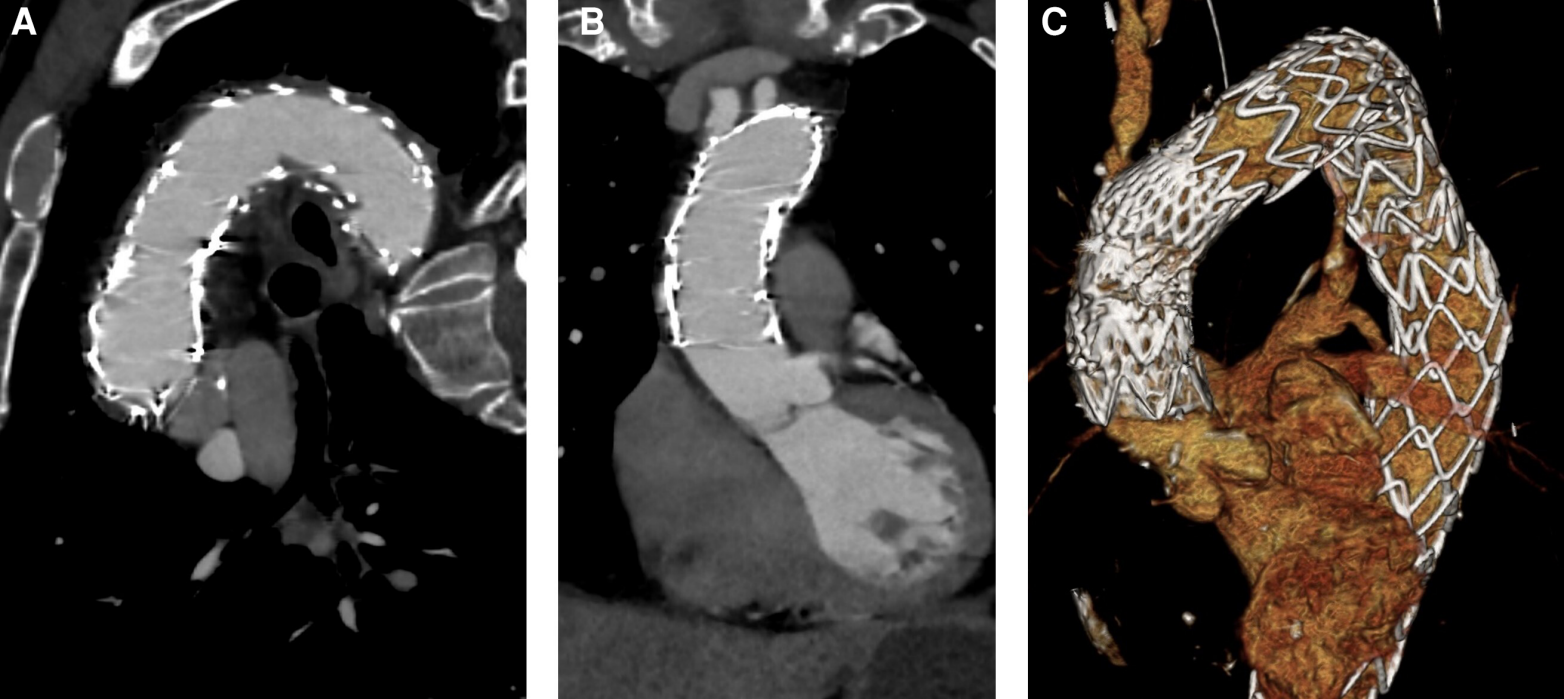
Postoperative CTA. (**A**) Stent grafts covering the ascending aorta and the aortic arch with no patent false lumen. (**B**) No residual dissection in the ascending aorta. (**C**) 3D reconstruction.

The patient was transferred to the intensive care unit under stable conditions. Due to slight anisocoria and still cold and pale right leg, CTA was performed. No signs of cerebral ischemia or vessel obliteration were evident. Right femoral and iliac artery was dissected, but distal perfusion was preserved and peripheral pulses were palpable. Symptoms diminished in the further course and the patient was extubated after the first postoperative day. However, he initially presented with paraparesis with only minimal preserved motoric function of the right leg. Fortunately, motoric function recovered significantly in the further course. No signs of endoleak or false lumen patency could be detected by CTA. Results of postoperative CTA (SOMATOM Definition Flash, Siemens®, Erlangen, Germany) is shown in Figure 4. The patient was referred to the normal ward on the eighth postoperative day after slowly recovering from postoperative delirium.

**Figure A1:**
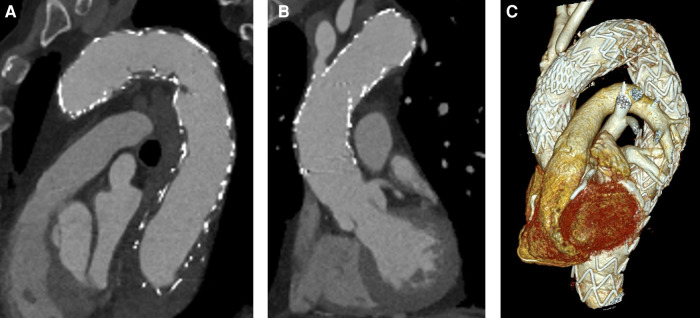
CTA follow-up 3.5 years later. (**A**) No endoleak in the aortic arch. (**B**) No disease progress in the ascending aorta. (**C**) 3D reconstruction.

**Figure A2:**
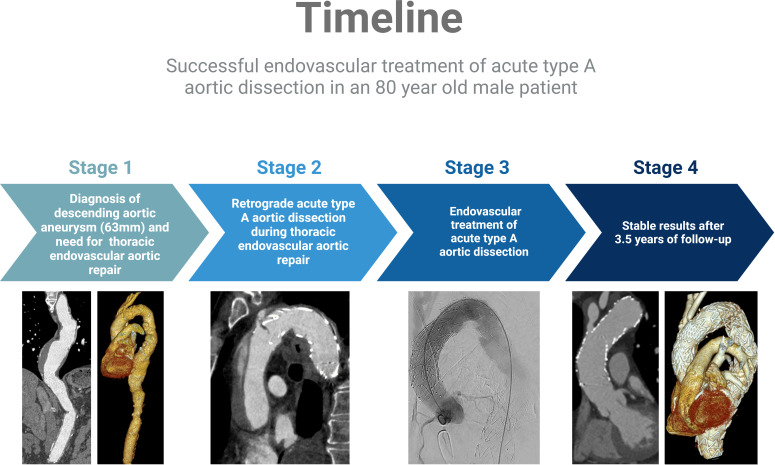
Timeline describing the patient's course including the relevant events.

No aortic re-interventions were necessary after discharge. After 3.5 years of follow-up, CTA showed no progress of the disease, including no evidence of stent failure, no signs of endoleak, and no aneurysm or dissection progress. CTA (SOMATOM Definition Flash, Siemens®, Erlangen, Germany) results are shown in Appendix Figure A1. A timeline that summarizes the patient's course including the relevant events is shown in Appendix Figure A2.

## 3. Discussion

In this case report, we demonstrate the efficacy and anatomical feasibility of an individualized endovascular approach for the treatment of ATAAD. Several other groups have reported similar cases, bearing in mind that these procedures were carried out in highly specialized aortic centers by an interdisciplinary team and require meticulous planning (12, 13). However, the rate of technical success is reported to be greater than 90%, and periprocedural mortality is extremely low (12–14). Thereby, the anatomical feasibility represents one of the key points and has been investigated in the past, proving that an endovascular approach may not only be restricted to rare, isolated cases (15). However, open surgical treatment still represents the gold standard of care and should be carried out, if possible (2). The overall aim to close/resect the entry tear to avoid aortic rupture and resolve organic malperfusion should be kept in mind, making no compromises (16). Especially due to the complex pathology of ATAAD including involvement of the aortic root, the coronary arteries, and additional branches of the aortic arch, endovascular approaches remain very controversial at this time. Most of the published data refer to cases with favorable anatomy and/or locally restricted disease processes, which significantly complicates the transfer of treatment concepts to more complex cases (17). Adding an uncovered nitinol stent for the aortic arch according to the PETTICOAT technique to minimize the risk of true lumen collapse and enhance the remodeling process of the dissected aorta is of upmost importance, but a rather individual approach according to this case (11). However, this “reverse PETTICOAT technique” may be a useful adjunct, especially considering recent results of the STABLE Trial that demonstrated favorable clinical and anatomical results as well as positive aortic remodeling when adding a distal bare metal stent (18). Of specific concern is the anchoring of the device in within the proximal landing zone (ascending aorta). Evolving concepts like the “Endo Bentall approach” published by the Freiburg group to address the challenges of aortic insufficiency and aortic root dissection are promising, but are still experimental (19). The choice of the stent graft as well as the degree of oversizing in the ascending aorta is one of the most difficult parts. In our case, proximal oversizing rate was 15%. Furthermore, available stent graft systems for the ascending aorta are scarce and restricted to off-label use, especially since Valiant Navion™ (Medtronic®, Dublin, Ireland) got removed from the market. Additional systems that can be used for the ascending aorta may be GORE® C-TAG® (GORE®, Flagstaff, AZ, USA), ZENITH TX2 (Cook, Bloomington, IN, USA), Relay NBS (Bolton, Barcelona, Spain; now Terumo®, Inchinnan, UK), and Lamed Ankura® (Lamed, Oberhachingen, Germany), wherein the latter may be used with caution due to the proximal extended stent tip (13). Different suggestions have been made for the minimum size of the landing zone in the ascending aorta and the safe distance to the coronary ostia, which vary between 10 and 40 mm (14). However, there is general agreement that new short-length stent graft devices are needed to address the anatomical challenges of the ascending aorta. Though we are able to demonstrate a successful endovascular approach for the treatment of complex ATAAD in DeBakey type 1 dissection with preoperative malperfusion, the aortic root including the aortic valve was not involved. Furthermore, no pericardial tamponade was evident, which would have been a major influencing factor for our strategy. These findings are in common with results from other groups (13). Therefore, concepts like the “Endo Bentall approach” are of particular interest for the future and may have great potential to treat elderly high-risk patients who are deemed to unfit for open surgery (19). Nevertheless, we are able to show stable results without any disease progress after 3.5 years of follow-up. In a multicenter series with 12 patients, Nienaber et al. could also detect no case of endoleak occurring during the whole follow-up period (13). These results are promising and demonstrate the potential sustainability of this approach for highly selected cases.

## Data Availability

The original contributions presented in the study are included in the article/Supplementary Material, further inquiries can be directed to the corresponding author.
